# The Therapeutic Effect of Zuogui Wan in Gestational Diabetes Mellitus Rats

**DOI:** 10.1155/2014/737961

**Published:** 2014-07-22

**Authors:** Yuwei Wang, Qianjin Feng, Xin Niu, Xinshe Liu, Kaixia Xu, Xiangzhu Yang, Huifeng Wang, Qiuju Li

**Affiliations:** ^1^Beijing University of Chinese Medicine, No. 11 North Third Ring Road, Chaoyang District, Beijing 100029, China; ^2^Shanxi University of Traditional Chinese Medicine, No. 89 Jinci road, Taiyuan, Shanxi 030024, China; ^3^Zhejiang University, Hangzhou, Zhejiang 310058, China

## Abstract

In this experiment, we established an animal model of gestational diabetes mellitus rats using streptozotocin. Using the rat model of GDM, the pregnant rats in 1-19d were divided into three groups: (1) Zuogui Wan gestational diabetes mellitus group (group I, *n* = 12), (2) gestational diabetes mellitus rats as the control group (group II, *n* = 11), and (3) rats of normal pregnancy group (group III, *n* = 11). Compared with gestational diabetes mellitus rats as the control group, Zuogui Wan can change the indexes of fasting blood glucose, body weight, total cholesterol, insulin, and metabolism cage index significantly in Zuogui Wan gestational diabetes mellitus group. We can conclude that Zuogui Wan has the therapeutic effect on gestational diabetes mellitus.

## 1. Introduction

Gestational diabetes mellitus (GDM) is a heterogeneous entity, including carbohydrate intolerance of variable severity with onset or first recognition during pregnancy [1]. With rapid development of the society, living standard has been greatly increased, which made a big change to people's diet, lifestyle, and work patterns. The prevalence of gestational diabetes mellitus increased rapidly, which puts a large burden on families and the society. A large scale blood screening survey of pregnant in American showed that the incidence of Asian women with gestational diabetes mellitus was 6.3% [2]. In China, a large scale blood screening survey of pregnant women showed that the incidence of Chinese women with gestational diabetes mellitus was 5.1%, with 7.1% incidence in the South of China and 3.9% incidence in the North of China [3]. Gestational diabetes mellitus seriously affect health of mothers and infants and glucose metabolism of offspring [4–6].

## 2. Materials

### 2.1. Experimental Animals

Experimental Wistar rats (female = 100, male = 100) are all adult rats with a body weight between 200 g and 250 g. The experimental animals were provided by Beijing Vital River Laboratory Animal Technology Co., Ltd (License number: SCXK (BJ) 2012-0001). The conduct of the experiments was in accordance with international zoology ethical standards.

### 2.2. Medicines and Reagent

Materials of Zuogui Wan (*Rehmannia* : yam :* Cornus* : wolfberry : dodder : antler : turtle  shell :* Cyathula* 8 : 4 : 4 : 4 : 4 : 4 : 4 : 3) were purchased from Beijing Tong Ren Tang and then verified by Medicine department of Beijing University of Chinese Medicine as genuine. We used ceramics to decoct and extract Chinese herbal medicines. The mass concentration is 1 g · mL^−1^ of dried herbs. Streptozotocin (STZ) was produced by American Sigma Corporation (Batch: B64927). We adjusted STZ dissolving solution to acidity (pH = 4.2) by 0.1 mol/L citric acid buffer solution, which was purchased from Beijing fraternity Port Company. Uric sugar test paper was purchased from Uritest Guilin Medical Electronic Sales Co., Ltd (Batch: 56130184). Chloral hydrate was purchased from Tianjin Fucheng Chemical Reagent Factory. Triglyceride detection kit was purchased from Beijing Wan Tai Derui Diagnostic Technology Co., Ltd (Batch: ZL2103AA02T). Cholesterol detection kit was purchased from Beijing Wan Tai Derui Diagnostic Technology Co., Ltd (Batch: ZL2105AA31).

### 2.3. Feed

A basic diet (ID = 1022) and high fat and sugar diet (protein 20%, carbohydrate 20%, and fat 60%) were all purchased from Beijing, China Fukang Biological Technology Co., Ltd (License number: SCXK (BJ) 2009-0008).

### 2.4. Instruments

Instruments used in this study included blood sugar detector (Johnson stable fold easily type LF033/V02), glucose test strips (Johnson Lot3354358), automatic biochemical analyzer (Toshiba TBA-40PR), rotary microtome, automatic dyeing machine, BX51 microscope, and biological automatic photographic image analysis system.

## 3. Methods

### 3.1. Model Preparation

*Model Preparation for Pregnant Rats*. 100 female rats were fed for one week under the condition of a temperature of 20–22°C, 30%~65% for relative humidity, 150~300Lx for illumination, and 12 : 12 day and night ratio. During the adaptive feeding period, saline (20 mL · kg^−1^) was administered to rats by gavage once a day. One week later, 100 sets of squirrels were fixed in shelves of hanging type. Meanwhile, a tray was placed under each squirrel. 100 female and 100 male rats were put together in cages in which the female/male ratios were 1 : 1. At the same time, two methods were used to examine the pregnant situation of female rats. Firstly, after 12 hours, the method of pessary in tray was used to observe whether there were pessaries (ivory and solid jelly). Then, rats with pessary in tray were taken to do vaginal smears. If sperm had been found by microscopic examination [4], then they were labeled as pregnant rats (0 d). A total of 34 pregnant rats were detected in this experiment. Then the pregnant rats were taken out, 23 were fed with high fat and sugar diet, 11 were fed with basic diet.

*Grouping and Treatment*. The 34 pregnant rats were divided into three groups: Zuogui Wan gestational diabetes mellitus group (*n* = 12), gestational diabetes mellitus as the control group (*n* = 11), normal pregnancy rats (*n* = 11). Both rats in Zuogui Wan gestational diabetes mellitus group and gestational diabetes mellitus as the control group were feed with high fat and sugar diet. Rats in normal pregnancy group were feed with basic diet. Zuogui Wan decoction (i.g., 1 g*·*mL^−1^, 20 mL*·*kg^−1^) was administered once a day to rats in Zuogui Wan gestational diabetes mellitus group by gavage for 19 days. Saline (20 mL · kg^−1^) was administered once a day to rats in gestational diabetes mellitus group and normal pregnancy group by gavage for 19 days.

*Establishment of Model of Rats of Gestational Diabetes Mellitus*. The 34 pregnant rats taken from the preparation stage (the first day of pregnancy) were fasted for 12 h. After that, 23 of them were injected peritoneally with dissolved STZ (40 mg/kg) and labelled as Zuogui Wan gestational diabetes mellitus group (*n* = 12) and gestational diabetes mellitus as the control group (*n* = 11) and the remaining 11 rats were labelled as normal pregnant group and received injection of sodium citrate buffer solution. 23 rats were given normal water and high fat and sugar diet after 4 h.The film forming standard of gestational diabetes was fasting blood glucose after 72 h of the injection of STZ ≥ 11.1 mmol/L or random blood glucose ≥16.7 mmol/L and urine glucose > ++. Rats of 4 days pregnancy in Zuogui Wan gestational diabetes mellitus group and gestational diabetes mellitus as the control group were all in accord with film forming standard of gestational diabetes mellitus.

### 3.2. Detection Index

*Observation of General Condition*. Fasting blood glucose and weight were observed and recorded in pregnancy, 0, 7, and 14 d. Meanwhile water intake, urinary output, feed intake, and fecal output were observed in pregnancy, 15 d. The rest of the indexes were detected after lactation.

*The Detection of Blood Lipid*. The serum TC and TG were detected by using enzyme-conjugated colorimetric analysis method by automatic biochemistry analyzer according to the procedures of kits.

*Insulin Determination*. Blood was drawn from abdominal artery from all rats. All the blood samples were centrifuged to get serum. The insulin level was detected by using enzyme-immunoassay method according to the procedures of kits.

*Pathomorphological Observation*. We took materials from pancreas. The pancreas was embedded by paraffin, and then we got the slice 4 *μ*m thick by routine paraffin section. Pathomorphological observation of pancreas collected for HE staining was examined by optical microscope.

### 3.3. Data Processing

Statistics work was completed with SPSS18.0 statistical software. Differences of measurement data were compared with one-way analysis of variance. *P* < 0.05 represented statistical significance. The comparison results were shown by notations as follows: ^∗/#^ *P* < 0.05; ^∗∗/##^ *P* < 0.01; ^∗∗∗/###^ *P* < 0.001.

## 4. Results

### 4.1. Observation of General Condition

Rats in normal pregnancy group during pregnancy had good mental status, they were flexible and very active, and they had shiny white hair and had normal feed intake, normal urine, and normal rat tail temperature. Rats in gestational diabetes mellitus as the control group were dizzy and unresponsive, had rough brown hair, and lost hair easily and had an increase in feed intake and water intake and a significant increase in urine output and lower tail temperature. Rats in Zuogui Wan gestational diabetes mellitus group were dysphoric and had shiny brown hair, cool tail, and less hair loss. The food intake, water intake, and urinary output have increased.

### 4.2. Impact of Zuogui Wan on Physiological Characteristics in Rats of Gestational Diabetes Mellitus

*Fasting Plasma Glucose and Body Weight*. As can be seen from Table 1, in the first 7 days of pregnancy, fasting plasma glucose was significantly higher (*P* < 0.001) in gestational diabetes mellitus as the control group and Zuogui Wan gestational diabetes mellitus group compared with normal pregnancy group. The means of fasting blood glucose in Zuogui Wan gestational diabetes group were lower than gestational diabetes mellitus as the control group, but the difference was not significant. In the 14th day of pregnancy, compared with gestational diabetes mellitus as the control group, fasting plasma glucose was significantly lower (*P* < 0.05) in Zuogui Wan gestational diabetes mellitus group. In terms of weight, in the 7th day of pregnancy, weight was significantly lower (*P* < 0.001, *P* < 0.01) in gestational diabetes mellitus as the control group and Zuogui Wan gestational diabetes mellitus group. Weight for gestational diabetes mellitus as the control group was lower in the first 7 days of pregnancy than in 0 days of pregnancy. In the first 14 days of pregnancy, compared with the normal pregnancy group, the weight of gestational diabetes mellitus as the control group reduced significantly (*P* < 0.01), but body weight in Zuogui Wan gestational diabetes mellitus group had no significant difference.

*Metabolism Cage Index*. As can be seen from Table 2, in the 15th day of pregnancy, compared with normal pregnancy group, water intake, urinary output, feed intake, and fecal output were all significantly higher (*P* < 0.001) in the control group and Zuogui Wan gestational diabetes group. Compared with gestational diabetes mellitus as the control group, water intake, urinary output, feed intake, and fecal output were all significantly lower (*P* < 0.001) in Zuogui Wan gestational diabetes mellitus group.

*Biochemical Index*. It is apparent in Table 3 that, compared with normal pregnancy group, triglyceride and insulin were all significantly higher (*P* < 0.001) in gestational diabetes mellitus as the control group and Zuogui Wan gestational diabetes mellitus group. Total cholesterol was significantly higher in gestational diabetes mellitus as the control group. Compared with gestational diabetes mellitus as the control group, total cholesterol and insulin were all significantly lower (*P* < 0.001) in Zuogui Wan gestational diabetes mellitus group.

**Figure 1:**
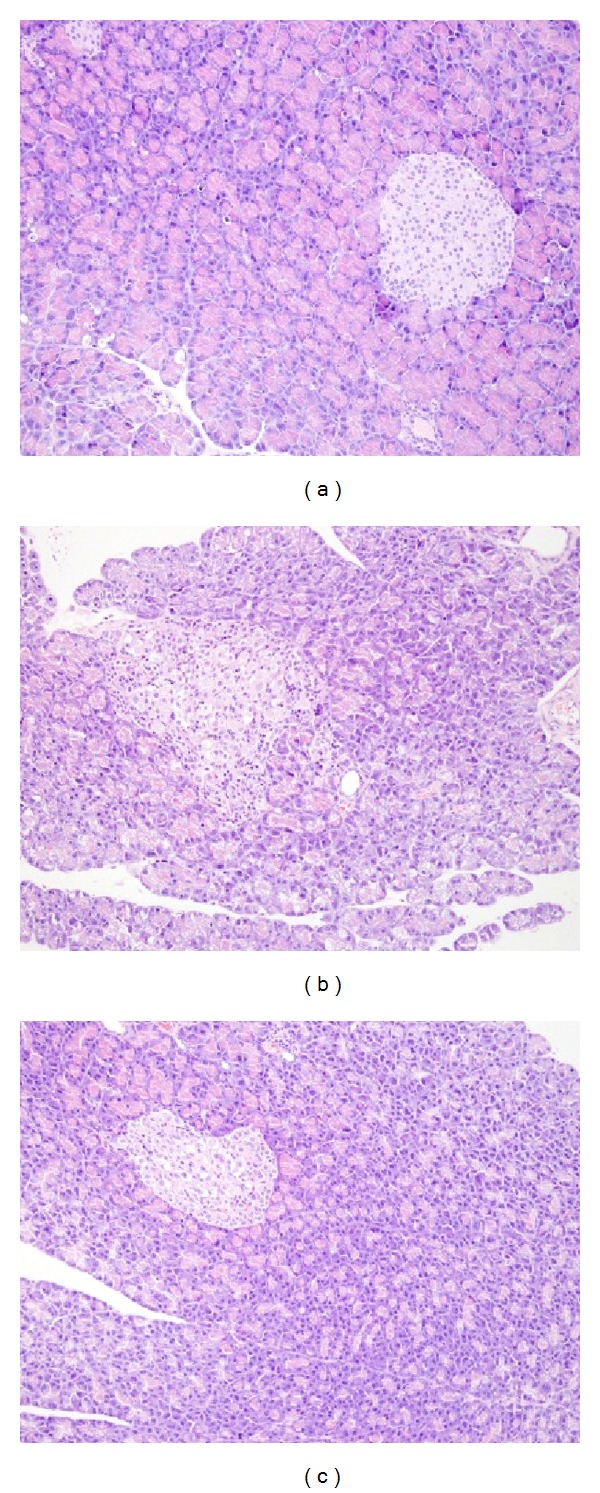
Comparison of pancreatic morphology of rats in three groups (HE, ×200). (a) Normal pregnancy group: normal pancreas. (b) Gestational diabetes mellitus as the control group: mild atrophy of islet, B cells moderate swelling, and interstitial inflammatory cells infiltration. (c) Zuogui Wan gestational diabetes mellitus group: pancreatic B cells swelled moderately and vacuolar degeneration occurred. The number of pancreatic B cells reduced slightly.

*Pathomorphological Observation*. It is clear from HE staining sections (Figure 1) that morphology and structure of pancreas were normal in normal pregnancy group. Rats in gestational diabetes mellitus as the control group had mild atrophy of islet; B cells swelled and vacuolar degeneration occurred; a little infiltration of inflammatory cells was found in some islets; pancreatic ductal revealed medium dilatation; appeared pancreatic acinar cells of some rats occurred vacuolar degeneration with mild-moderate interstitial inflammatory cells infiltration. Rats in Zuogui Wan gestational diabetes mellitus were mild atrophy of islet; B cells had reduced and a few of pancreatic ducts revealed A medium dilatation which lead to some inflammatory cell infiltrations around.

Graduation statistics method had been given to pancreas in each group according to pancreatic endoscopic diagnostic criteria in Table 4. Compared with gestational diabetes mellitus as the control group, pancreatic lesions in Zuogui Wan gestational diabetes mellitus group reduced significantly (*P* < 0.05) (Table 5).

## 5. Discussion

There are two kinds of model establishment methods of rats in gestational diabetes mellitus. One method is to prepare animal diabetes model in pregnant rats by intravenous injection of alloxan, which was proposed by Yang et al. [7]. As alloxan is poisonous to B cell and unstable, therefore in this study, we made a reference of another GDM proposed by Liu et al. [8], which is established by using streptozotocin. Compared with the alloxan's model, the symptoms by streptozotocin's model of diabetic ketoacidosis and serum free fatty acid are ease, and this model helped us achieve the required results of the experiment. The experiments of Wang et al., [9] confirmed that* Rehmannia glutinosa* oligosaccharides can reduce the blood glucose of gestational diabetes mellitus rats and improve the insulin release. The experiments of Qiu and Li [10] confirmed that* Ophiopogon japonicus* polysaccharide can reduce the blood glucose of gestational diabetes mellitus rats.

Zuogui Wan was first introduced in volume Fifty-one of Jing Yue Quan Shu. It is composed of Big Huai* Rehmannia *240 g, yam 120 g (frying), medlar (120 g),* Cornus *120 g, Chuanniuxi 120 g (wine washing and steam mature), dodder 120 g, deer adhesive 120 g (chopping and frying), and glue of tortoise plastron 120 g (chopping and frying). The efficacy of Zuogui Wan is nourishing Yin, tonifying the kidney, replenishing essence, and nourishing marrow. Modern pharmacological studies suggest that catalpol,* Rehmannia glutinosa* oligosaccharides and stachyose in* Rehmannia* has hypoglycemic effect [11–13]; doctors often use yam to treat XiaoKeBing; diosgenin and mannan in yam has hypoglycemic effect [14, 15]; wolfberry polysaccharide in medlar has hypoglycemic effect [16]; ursolic acid in* Cornus* has hypoglycemic effect [17]; Chuanniuxi, dodder, deer adhesive, and glue of tortoise plastron have hypoglycemic effect. Zuogui Wan is composed of the eight kinds of Chinese herbal medicines. So it has a strong hypoglycemic effect and can improve the body immunity efficiently. Experimental study of Yu et al. [18] shows that Zuogui Wan has hypoglycemic effect on the experimental diabetes mellitus rats.

The mechanism of Zuogui Wan reducing blood glucose in diabetic pregnant rats may have a relationship with the fact that the effect of Zuogui Wan can adjust hypothalamic-pituitary-adrenal axis. The balance of hypothalamic-pituitary-adrenal axis in gestational diabetes mellitus rats was destroyed. Zuogui Wan can recover the balance of adrenal axis of gestational diabetes mellitus rats. The experiments of Liu et al. [19] confirmed that Zuogui Wan can significantly improve retardation caused by hypothalamic arcuate nucleus damaged by monosodium l-glutamate and abnormal proliferation reaction of thymocytes and lymphocytes cells. Gao [20] used Zuogui Wan to treat type 2 diabetic nephropathy. High blood pressure patients took conventional hypoglycemic drugs and at the same time took Zuogui Wan 6 g, 2 times a day. The total effective rate was 78.15%. Feng et al. [21] demonstrated that “Zuogui Wan drugs serum” has a significant role in promoting and improving zygote division of rats using technology of mouse fertilized in vitro. Xu et al. [22] confirmed that using Zuogui Wan in embryonic development of rats can promote intrauterine fetal development and the treatment of intrauterine growth retardation and can make the immune function of adult male offspring better.

What can be seen from the results of this experiment is that Zuogui Wan can reduce fasting blood glucose and maintain a stable growth speed of body weight in rats of gestational diabetes mellitus when Zuogui Wan was given to pregnant rats. Compared with gestational diabetes mellitus as the control group, the means of blood glucose of rats in Zuogui Wan gestational diabetes mellitus group in the first 14 days of pregnancy decreased, but a gap still existed compared with normal blood glucose of rats in normal pregnancy group. Compared with gestational diabetes mellitus as the control group, water intake, urinary output, feed intake, and fecal output were all significantly lower (*P* < 0.001) in Zuogui Wan gestational diabetes mellitus group. The results proved that Zuogui Wan can significantly relieve symptoms of diabetes when given in embryonic rats of gestational diabetes mellitus.

There was no significant difference in total cholesterol between Zuogui Wan gestational diabetes mellitus group and normal pregnancy group. Compared with gestational diabetes mellitus as the control group, total cholesterol and insulin were significantly lower (*P* < 0.001) in gestational diabetes mellitus group. Therefore, it is clear that Zuogui Wan can reduce the level of total cholesterol and insulin when given in embryonic rats of gestational diabetes mellitus. But a gap still existed compared with normal pregnancy group.

Chinese medicine believes that patients with diabetes often have some characteristics like “Yang is usually excessive while Yin is frequently deficient.” Maternal gestation period needs blood filling. Gestational diabetes rats must have characteristics of Yin which is often inadequate. The efficacy of Zuogui Wan is nourishing Yin and tonifying the kidney, replenishing essence, and nourishing marrow. Thus, it has a therapeutic effect on the rats of gestational diabetic, but a gap still exists compared with normal pregnancy group. There are two possible reasons to explain this phenomenon. On the one hand, sample size of this study is small. On the other hand, high fat and sugar diet might have some uncontrollable effects. Body weight of rats in gestational diabetes mellitus group grew slowly in this study, which might be attributed to the loss of large quantity of sugar, protein, and some of the nutrients through urine during pregnancy. What is more, Zuogui Wan can maintain a stable growth speed of body weight in rats of gestational diabetes mellitus group when Zuogui Wan was given to pregnant rats. This study provides experimental evidence for the fact that “kidney is responsible for growth, development, and reproduction” and acts as an important basis for later studies of gestational diabetes mellitus.

## Figures and Tables

**Table 1:** Impact of Zuogui Wan on fasting blood glucose and body weight in rats of gestational diabetes mellitus.

Group	Fasting blood glucose (mmol*·*L^−1^)			Body weight (g)		
	GD0	GD7	GD14	GD0	GD7	GD14
Normal pregnancy group (11)	5.32 ± 0.63	5.5 ± 0.55	5.29 ± 0.63	236.32 ± 18.58	263.16 ± 23.54	293.37 ± 30.73
Gestational diabetes mellitus as the control group (11)	5.11 ± 0.70	15.92 ± 4.39***	16.92 ± 8.65***	232.47 ± 12.61	228.32 ± 23.25***	248.67 ± 42.68**
Zuogui Wan gestational diabetes mellitus group (12)	5.23 ± 0.68	13.62 ± 3.55***	11.71 ± 5.55^∗#^	234.99 ± 16.50	237.55 ± 16.05**	264.82 ± 33.15

Note: all results are presented as mean ± S.D. ∗indicates a significant difference compared with the normal pregnancy group. ^#^indicates a significant difference compared with the gestational diabetes mellitus as the control group. ^∗/#^ *P* < 0.05; ^∗∗/##^ *P* < 0.01; ^∗∗∗/###^ *P* < 0.001.

**Table 2:** Impact of Zuogui Wan on metabolism cage index in rats of gestational diabetes mellitus.

Group	Water intake (mL/kg/day)	Urinary output (mL/kg/day)	Feed intake (g/kg/day)	Fecal output (g/kg/day)
Normal pregnancy group (11)	139.18 ± 9.47	66.54 ± 9.98	73.09 ± 13.46	17.55 ± 4.70
Gestational diabetes mellitus as the control group (11)	761.18 ± 93.64***	638.82 ± 77.90***	159.82 ± 32.55***	85.18 ± 20.64***
Zuogui Wan gestational diabetes mellitus group (12)	290.50 ± 90.50^∗∗∗###^	202.83 ± 79.57^∗∗∗###^	106.33 ± 29.08^∗∗###^	49.42 ± 14.61^∗∗∗###^

Note: all results are presented as mean ± S.D. ∗indicates a significant difference compared with the normal pregnancy group. ^#^indicates a significant difference compared with the gestational diabetes mellitus as the control group. ^∗/#^ *P* < 0.05; ^∗∗/##^ *P* < 0.01; ^∗∗∗/###^ *P* < 0.001.

**Table 3:** Impact of Zuogui Wan on biochemical index in rats of gestational diabetes mellitus.

Group	Triglyceride (mmol*·*L^−1^)	Total cholesterol (mmol*·*L^−1^)	Insulin (mIU*·*L^−1^)
Normal pregnancy group (11)	0.49 ± 0.11	1.27 ± 0.37	7.68 ± 0.32
Gestational diabetes mellitus as the control group (11)	0.94 ± 0.16***	2.02 ± 0.38***	9.35 ± 0.42***
Zuogui Wan gestational diabetes mellitus group (12)	0.89 ± 0.18***	1.32 ± 0.26^###^	8.44 ± 0.55^∗∗∗###^

Note: all results are presented as mean ± S.D. ∗indicates a significant difference compared with the normal pregnancy group. ^#^indicates a significant difference compared with the gestational diabetes mellitus as the control group. ^∗/#^ *P* < 0.05; ^∗∗/##^ *P* < 0.01; ^∗∗∗/###^ *P* < 0.001.

**Table 4:** Pancreatic endoscopic diagnostic criteria.

Grade	Lesions condition of pancreas
−	Normal morphology and structure of pancreas
+	Inflammatory cell infiltration, B cells swelled and occurred vacuolar degeneration; pancreatic acinar cells of some rates occurred vacuolar degeneration, mild interstitial inflammatory cells infiltration; pancreatic ductal revealed mild dilatation
++	Pancreatic B cells particles depigmentation, swelling and occurred vacuolar degeneration, mild atrophy of islet; the number of pancreatic B reduced slightly

**Table 5:** Comparison of pancreatic lesions of rats in each group.

Group	*N*	Pancreatic lesions			Rank sum test Rank values
		−	+	++	
Normal pregnancy group	11	11	0	0	7.50
Gestational diabetes mellitus as the control group	11	1	2	8	24.77
Zuogui Wan gestational diabetes mellitus group	12	2	6	4	20.00
